# Enhanced Methods for Needle Biopsy and Cryopreservation of Skeletal Muscle in Older Adults

**DOI:** 10.37421/jch.2020.11.553

**Published:** 2020-04-10

**Authors:** Cathy C Lee, Austin Hoang, David Segovia, Allen Herbst, Florian Barthelemy, Elizabeth Gibbs, Rachelle Crosbie, Stanley F Nelson, Carrie Miceli, Jonathan Wanagat

**Affiliations:** 1 VA Greater Los Angeles, Department of Veterans Affairs, Los Angeles, CA, USA; 2 Division of Geriatrics, Department of Medicine, David Geffen School of Medicine at UCLA, Los Angeles, CA, USA; 3 Department of Agricultural, Food and Nutritional Sciences, University of Alberta, Edmonton, Alberta, Canada; 4 Center for Duchenne Muscular Dystrophy, University of California, Los Angeles, Los Angeles, CA, USA; 5 Department of Microbiology, Immunology, and Molecular Genetics, David Geffen School of Medicine and College of Letters and Sciences, University of California, Los Angeles, Los Angeles, CA, USA; 6 Department of Integrative Biology and Physiology, University of California, Los Angeles, CA, USA; 7 Molecular Biology Institute, University of California, Los Angeles, Los Angeles, CA, USA; 8 Department of Neurology, David Geffen School of Medicine, University of California, Los Angeles, USA; 9 Department of Human Genetics, David Geffen School of Medicine, University of California, Los Angeles, Los Angeles, CA, USA; 10 Department of Pathology and Laboratory Medicine, David Geffen School of Medicine, University of California, Los Angeles, Los Angeles, CA, USA

**Keywords:** Skeletal muscle, Aging, Biopsy, Cryopreservation, Histology

## Abstract

Human muscle biopsies are increasingly important for diagnosis, research, and to monitor therapeutic trials. We examined the use of a self-contained, vacuum-assisted biopsy system and a novel muscle freezing technique to improve, simplify, and standardize human muscle biopsy collection and cryopreservation in older adults.

The VACORA vacuum-assisted biopsy system was deployed in muscle biopsies of 12 individuals ranging in age from 57 to 80 years. This office-based approach was well tolerated as it is minimally invasive, uses only local anesthetic, and has a quick recovery. To maximize biopsy sample quality and reproducibility, we developed a novel muscle sample freezing protocol. Fresh muscle biopsy samples were placed into readily available tissue cassettes followed by direct freezing in liquid nitrogen. After this modified snap freezing protocol, frozen muscle samples were enrobed in embedding medium for cryosectioning. We examined the effect of this freezing approach in histological sections of rodent and human muscle samples.

The VACORA Biopsy System provided as many as four skeletal muscle core samples from a single biopsy site. Biopsy samples from 12 older adults weighed an average of 147.5 ± 11 mg each and had a consistent size and shape. There were no complications, and the residual scar is less than 10 mm. The freezing method using standard tissue cassettes with direct freezing in liquid nitrogen yielded high quality cryopreserved muscle tissue suitable for histological analysis without the need for isopentane and with little to no freeze-thaw damage.

These enhancements have streamlined and improved the consistency of our muscle biopsy protocol and provide sufficient high-quality sample for multi-dimensional downstream studies of human muscle in aging and disease.

## Introduction

The worldwide prevalence of neuromuscular disease is growing and now approximates that of other diseases such as Parkinson’s [1]. Diagnosis, basic research, and therapeutic trials will benefit from ready access to human skeletal muscle samples from a wide range of subjects. Diagnosis of neuromuscular disorders often relies on clinical observations, but is accelerated with molecular studies of the muscle itself. Older adults may have muscle biopsies to investigate acute muscle weakness, but the unrelenting age-induced declines in muscle mass or function rarely prompt a muscle biopsy. This is despite our current lack of a clear etiology for muscle aging. The advent of therapeutic interventions for some neuromuscular diseases requires validated biomarkers of treatment efficacy.

There is a growing interest in tissue and cellular heterogeneity, qualities that can only be assessed in primary tissue samples. Skeletal muscle is no exception as many pathological changes in muscle are characteristically focal or segmental e.g., fiber loss, fiber type switching, fibrosis, and group atrophy [2]. Muscle stem cells are another focal phenomenon in skeletal muscle that rely on an understanding of the *in vivo* niche and, therefore, require primary tissue samples [3].

**Figure 1.**
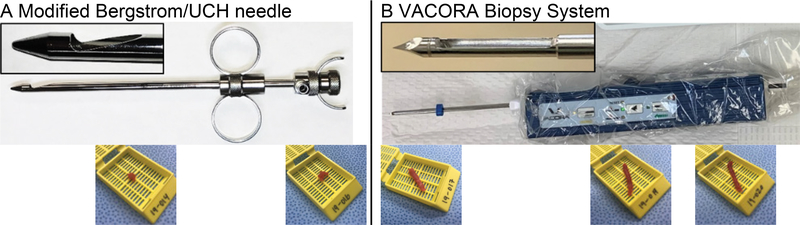
Comparison of Bergstrom and VACORA biopsy needles and representative muscle samples A- Bergstrom/UCH needle. Inset shows sample window that is 12 mm long from the 6-gauge needle. Bottom row of images shows consecutive representative muscle samples taken with the modified Bergstrom needle (cassette dimensions are 2.5W × 3L × 0.5D cm). B- VACORA self-contained, vacuum-assisted biopsy system in sterile overwrap. Inset shows sample window that is 15 mm long from the 10-gauge needle. Bottom row of images shows consecutive representative muscle samples taken with the VACORA Biopsy System (cassette dimensions are the same as in Panel A).

In the standard open muscle biopsy, the typical amount of muscle collected is approximately (5 to 550 mg and sizes range from 0.5 × 1 cm to 5 × 2 cm) [4]. This procedure is costly, requires an operating room, specially qualified personnel, and general or regional anesthesia, which may be of greater risk in younger and older individuals. The procedure and recovery times are lengthy and results in a considerable scar (e.g., ~30 mm) [4]. All of the above generally limit open biopsies to a single site in a single muscle and decrease the likelihood of later biopsies. A common alternative to open biopsy, particularly in the research setting, is needle biopsy using the Bergstrom or vacuum-enabled University College Hospital (UCH) needles (Figure 1). These needles allow for the use of local anesthetic in an office-based setting for the sampling of small amounts of human muscle. The sample size for the Bergstrom needle is typically ~100 mg, but size can vary widely with operator skill [5–7]. Recovery time from the needle biopsy is generally within a day with only a 1 cm scar remaining. The challenges of Bergstrom needle biopsy include the technical difficulty, lack of sample consistency, and small sample size.

An additional challenge with human muscle biopsies, and muscle histological studies in general, is the effect of freeze/thaw damage on microscopic tissue morphology. It is thought that slow freezing of muscle tissue allows formation of ice crystals that disrupt the muscle fiber architecture in subsequent histological preparations. Prevention of freeze/thaw damage in muscle typically focuses on freezing muscle samples as quickly as possible. Liquid nitrogen (−170 Celsius) is the coldest available freezing substrate and is readily available in research facilities and clinical offices (where it is used by dermatologist for ablative procedures on the skin). The freezing rate of an object in liquid nitrogen is hampered by the formation of a gaseous layer of evaporated liquid nitrogen that surrounds the object. The gaseous layer effectively insulates the object from the liquid nitrogen and reduces heat transfer. To circumvent this, muscle samples are often frozen in a liquid nitrogen-chilled isopentane, an organic solvent with a freezing temperature of −160 celsius. Because it does not form a gaseous layer around the sample, the isopentane transfers heat from the sample more quickly. Isopentane is not readily available, particularly in clinical settings, has a low flash point, and is difficult to maintain at the correct temperature when chilled in liquid nitrogen. In settings where large numbers of samples need to be processed quickly (e.g., mouse muscle harvest at a specific time point) or in clinical setting, the use of isopentane can be prohibitive. An ideal freezing protocol for muscle samples would: 1) optimize histological quality with the avoidance of freeze damage; 2) avoid the use of specialized reagents or equipment that are not readily available or have safety issues; 3) facilitate correct tissue orientation and sufficient embedding material to facilitate serial sections transverse to the long axis of the muscle fibers.

To simplify and improve the consistency of our human muscle biopsy protocol in older adults, we have deployed a self-contained, vacuum-assisted biopsy device (i.e., the VACORA Biopsy System) and developed a novel freezing protocol that uses a tissue cassette and freezing of samples directly in liquid nitrogen. The VACORA System greatly improves the amount, quality and consistency of the older human muscle biopsies while the new muscle freezing method is quicker and provides high quality histology. Combined, these approaches have broad potential utility in expanding skeletal muscle aging research by providing consistently high quality primary human tissues.

## Methods

### Animal muscle samples

Wild-type mouse (C57BL/6J), *mdx* dystrophin-null mouse (Jackson Labs C57BL/10ScSn-Dmdmdx/J) and rat (F344 × BN F1 hybrid) tissues were harvested from rodents maintained following guidelines established by the Institutional Animal Care and Use Committee at the University of California, Los Angeles and University of Alberta, Edmonton. Tissues were dissected and frozen as described below.

### Human muscle biopsies

Human muscle biopsies were obtained with informed consent from patients of the UCLA Center for Duchenne Muscular Dystrophy (CDMD) under University of California Los Angeles IRB-approved protocols #11–001087 and #18–001547. Skeletal muscle biopsies from the vastus lateralis were performed following a previously described protocol (5) except that a VACORA Biopsy System (Bard) with a 10-gauge needle was used instead of a Bergstrom needle. The VACORA biopsy system is a self-contained, vacuum assisted biopsy system (Figure 1B). From 2–4 biopsy cores were taken at each biopsy session through a single 3–4 mm skin incision. Muscle biopsy cores were frozen as described below and stored at −80 celsius.

### Histological processing of muscle samples

**Figure 2.**
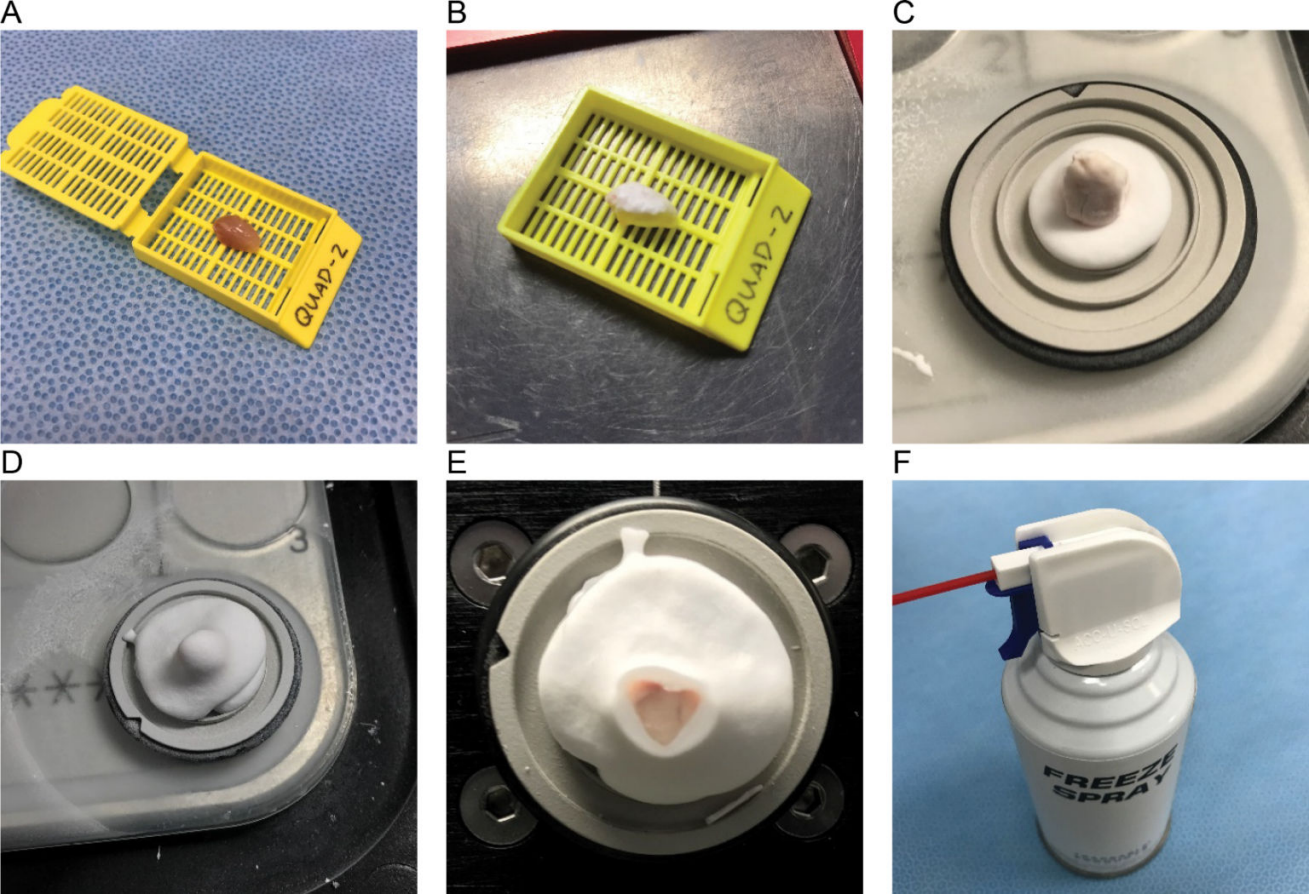
Steps in freezing and embedding protocol using cassettes directly in liquid nitrogen A- Mouse quadriceps muscle in labelled cassette. B- Frozen mouse quadriceps after 10 seconds of immersion in liquid nitrogen. C- Frozen mouse quadriceps mounted on chuck. D- Mounted mouse quadriceps enrobed in OCT. E- Faced off mouse quadriceps ready for serial cryosectioning. F- Freeze spray used to keep tissue from thawing during mounting and enrobing.

Muscle samples were placed into labeled 2.5 × 3 × 0.5 cm or 2.5 × 3 × 1 cm SWINGSETTE™ M515 Tissue Processing and Embedding Cassettes (Thermo Fisher Scientific) depending on the sample size and the cassette securely closed (Figure 2). Cassettes with sample were then immediately plunged into liquid nitrogen with a 12 inch forceps and held there until vigorous bubbling ceased (approximately 10 seconds). Frozen samples were removed from the cassette and either immediately mounted for cryosectioning or placed in a Thermo Scientific™ Nunc™ Biobanking and Cell Culture Cryogenic Tube and frozen at −80 C until later processing. Enrobing and mounting of samples on the cryostat sample holder, while keeping the samples frozen, was facilitated using Freeze Spray (Thermo Fisher Scientific) and prechilled Optimal Cutting Temperature media (Sakura). Samples were sectioned at 10-micron thickness onto ProbOn Plus microscope slides (Thermo Fisher Scientific) and stained with standard protocols with hematoxylin and eosin to asses freeze/thaw damage. Digital histological images were acquired using standard light and fluorescent microscopy and processed with Axiovision software (Zeiss) and/or ImageJ software.

### Statistical analysis

All biopsy sample weight data were presented as means ± SEM.

## Results

### VACORA needle biopsies in older adults

Figure 1 demonstrates the differences between the traditional Bergstrom needle and the VACORA Biopsy System used in this study. The Bergstrom needle has a sample window of 12 × 5 mm while the VACORA 10-gauge needle used in this study has a sample window measuring 3 × 20 mm. The VACORA needle also has an optional outer cannula that can be left in place between acquiring individual cores to maintain the needle track. The tip of the VACORA needle is considerably sharper than the Bergstrom needle (Figure 1), which allows for easier insertion of the VACORA needle through subcutaneous tissue, muscle fascia, and muscle. We have found that nicking the fascia is not required with the VACORA needle. The depth gauge on the VACORA needle allows the user to re-introduce the sample window to the same or different depths in the muscle or subcutaneous tissue as needed. We found this important to ensure that muscle was sampled rather than subcutaneous fat and aided in avoiding biopsies of the muscle fascia.

The VACORA system is built around a self-contained, sterile vacuum system. This system activates after the sample window opens to draw in surrounding tissue before the windows closes. As a self-contained vacuum system, it is able to pull a more consistent vacuum than the Bergstrom needle, and requires only a thumb push by the biopsy performer to activate. A vacuum may be applied to the Bergstrom needle via a syringe connected to the Leuer lock on the inner cutting cannula, the various interlocking parts of the Bergstrom needle (outer cannula, inner cutting cannula, and inner stylet) and differences between operators often prevent generation of a suitable vacuum.

We have deployed the VACORA Biopsy System for muscle biopsies in subjects with ages ranging from 57 to 80 years of age. We focused on older adults because of our ongoing studies of muscle aging. The procedure has been well tolerated, with 2–4 muscle biopsy cores taken per session from a single biopsy site of the vastus lateralis. The dimensions and mass of each core are consistent between biopsies with an average mass across all biopsies of 147.5 ± 11 mg per core. This provided a total of 288 to 576 milligrams of muscle tissue per biopsy session per subject. A typical needle core is approximately 15 mm in length and 3 mm in diameter. Fiber direction is readily determined from macroscopic inspection and enables proper tissue orientation during freezing, embedding, and cryosectioning.

### Tissue cassettes facilitate a liquid nitrogen-only freezing method

To improve simplify the freezing of muscle samples while maintaining excellent tissue quality, we have adapted a method of direct freezing in liquid nitrogen [8]. Figure 2 outlines the steps of this freezing protocol. Use of the fenestrated tissue cassettes promotes the flow of liquid nitrogen around the tissue sample, which is thought to reduce the insulating vapor barrier that slows heat transfer (i.e., the Leidenfrost effect), and increase the rate of tissue freezing. The tissue cassettes have the added benefits of being easy to label for sample identification, are commonly available, inexpensive, and disposable. The use of a freeze spray helps to keep the sample frozen during subsequent handling.

**Figure 3.**
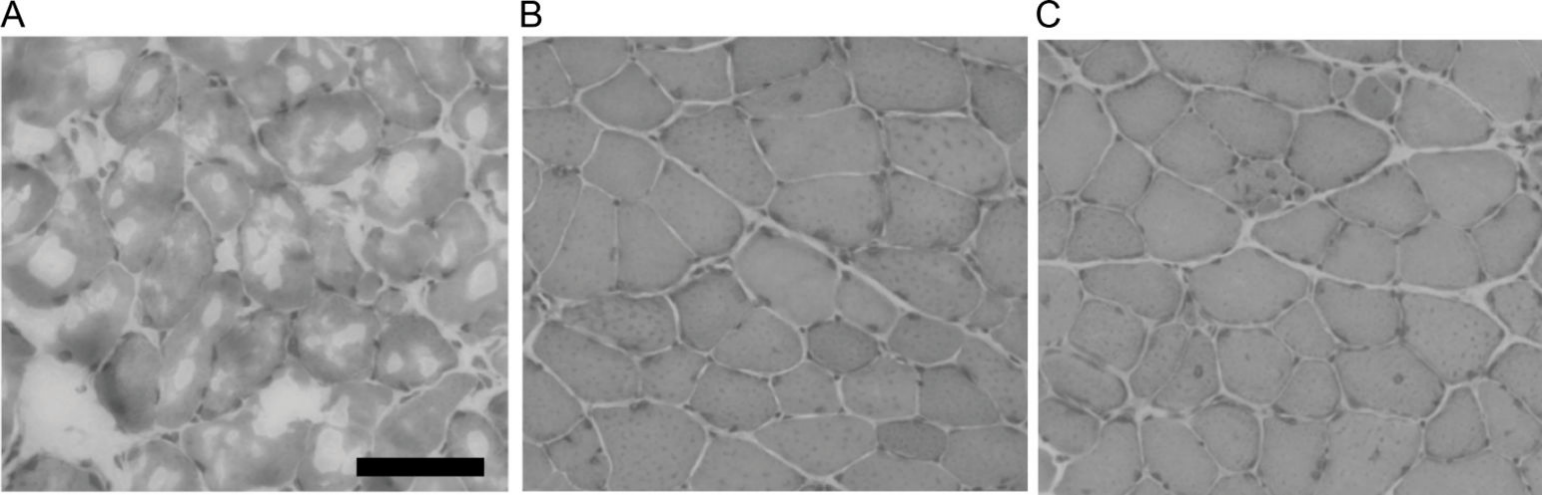
Comparison of muscle cryopreservation methods. All panels show mouse quadriceps muscle. A- Dry ice. B- Liquid nitrogen-chilled isopentane. C- tissue cassette directly into liquid nitrogen. The black bar in A is 100 microns.

**Figure 4.**
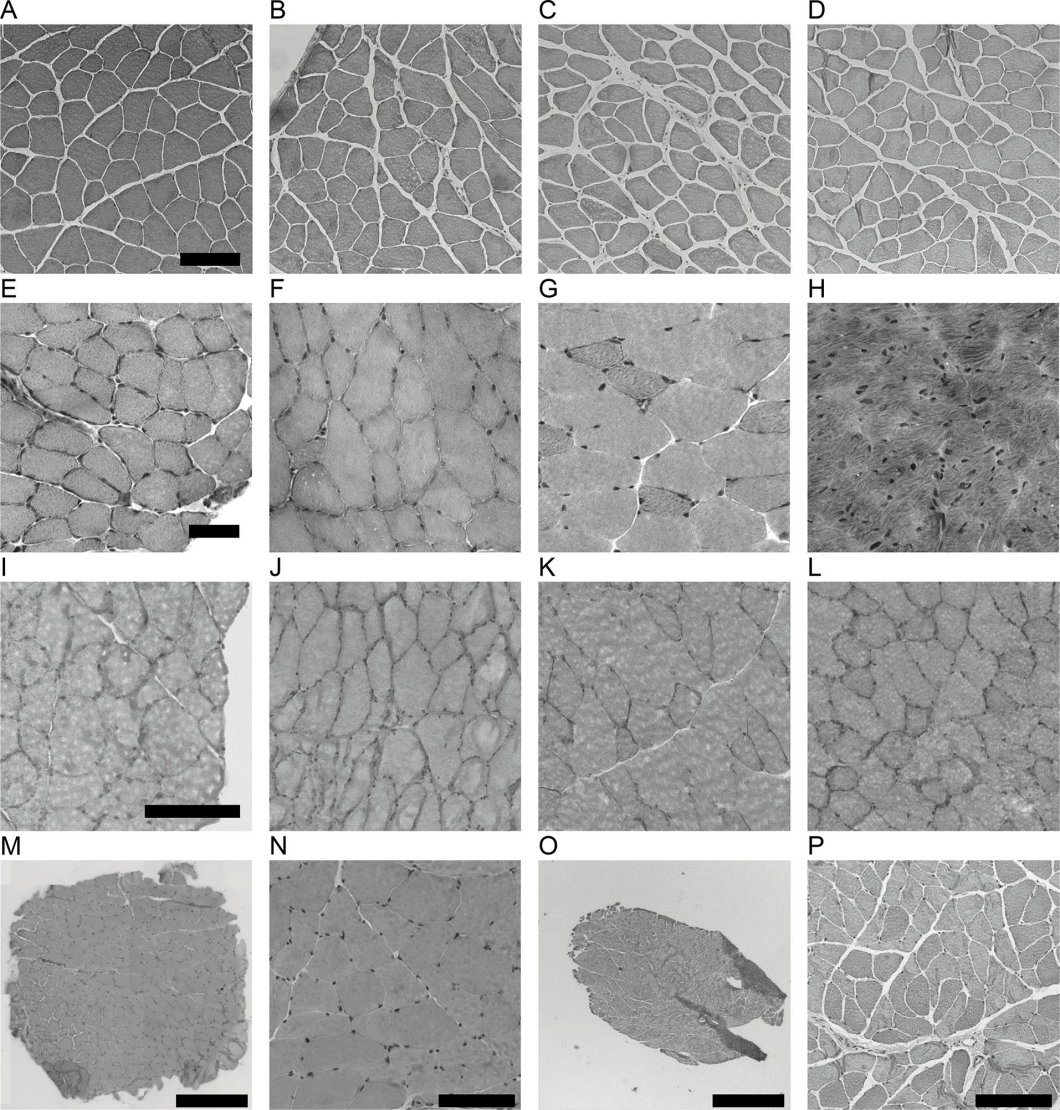
Quality of histological sections from muscle and biopsies following cassette freezing directly in liquid nitrogen. A through H are H&E stained cryosections of mouse tissues. A through D are sections from four individual mouse quadriceps muscles. E is soleus muscle. F is tibialis anterior muscle. G is extensor digitorum longus. H is heart tissue. I through L are four individual rat quadriceps muscles. M through P are cryosections from human vastus lateralis biopsies. M low magnification image of whole biopsy section stained with H&E from a 57 year old female subject. N- high magnification of M. O is a low magnification image of a whole biopsy section stained with H&E from a 64 year old subject. P- high magnification of O. Scale bar in A is 100 microns, E is 50 microns, I is 100 microns, M and O are 1000 microns, N and P are 100 microns.

Using this approach, we examined the quality of tissue cryopreservation in individual samples of five mouse muscles (quadriceps, soleus, tibialis anterior, extensor digitorum longus, and heart) old rat quadriceps, and human muscle core biopsies as shown in Figures 3 and 4. We first used rodent tissues to verify this method before applying to more precious human muscle biopsy samples. We observed little to no freeze/thaw damage in the mouse (Figure 4A–4H), normal healthy young and old human muscle samples (Figure 4M–4P). Using the VACORA system preserves biopsy orientation and thus clean cross-sections are obtained without the need to reposition the sample during cryosectioning. We observed only mild freeze thaw damage in the old rat quadriceps samples, which are ~10-fold larger than VACORA collected human biopsy or mouse quadriceps samples.

## Discussion

We have instituted a muscle biopsy and sample freezing protocol that has enhanced our research throughput, simplified our protocols, and improved sample quality and consistency. The VACORA Biopsy system allows office-based muscle sampling from a broad range of subjects. As with other needle biopsy approaches, the VACORA Biopsy System only requires local anesthetic (lidocaine) and has low complication rates. Because of the relatively low pain of the procedure, most patients have been willing to allow repeat biopsies. In our hands, the procedure takes 20–30 minutes per patient depending on the number of cores taken (i.e., typically four cores per muscle). Benefits of the VACORA System over the Bergstrom needle include consistent muscle biopsy core size, shape, orientation, and mass despite the fact that the VACORA needle is substantially narrower (i.e.,10-gauge vs 6-gauge).

As an invasive procedure, it is important that these precious samples can be utilized to their fullest extent. The size of each VACORA biopsy core and the ability to take several cores from the same skin incision from the vastus lateralis provides tissue amounts that rival those obtained by open biopsy. For our studies, the VACORA system provides enough muscle for histological, genetic, biochemical, and cell culture experiments from a single biopsy site and session.

Our experience with the VACORA Biopsy System mirrors others who have applied this device to human muscle biopsies. A VACORA System with a smaller 16-gauge needle was used in a diabetes mellitus study [9]. In this study, the vastus lateralis muscles of 57 subjects either with or without diabetes mellitus were biopsied for use in RNA isolation and gene expression studies. The average biopsy time in this study was 12 minutes and there were no complications. A more recent study examined the use of the VACORA System for diagnosis of neuromuscular diseases [10]. This study reviewed 102 VACORA biopsies in individuals with a mean age of 55 and found a mean sample weight of 190 milligrams. The complication of intramuscular hematoma in that study was 3/102 (3%), which they felt could be due to the more vigorous suction provided by the VACORA System, or the sharper needle tip.

The increased availability and consistency of muscle biopsy core samples using the VACORA System necessitated the search for methods that would increase sample freezing throughput and consistency. We found a protocol espousing the use of a specialized cryovial designed to promote flow of the liquid nitrogen around the sample and prevent the formation of a gaseous layer that inhibits freezing (the Leidenfrost effect) [8]. This multi-hole cryovial is not commercially available, so we adapted a commonly used tissue cassette. Tissue cassettes are routinely used in formalin fixation and paraffin embedding of tissues. The fenestrated sides of tissue cassette promote the flow of liquid nitrogen around the sample as is the case with the multi-hole cryovial. Our data show no freezing artefact in mouse or human biopsy muscle samples and only mild artefact in the rat quadriceps. The mild freeze/thaw artefact in the rat quadriceps samples likely arises from the relatively larger size of the tissue (~1 grams), which slows freezing. Added benefits of the tissue cassettes for direct freezing of samples in liquid nitrogen include ease of labeling and a variety of size to accommodate different tissues. Further, the liquid nitrogen freezing in cassettes improved our ability to process several biopsies as the issue of maintaining the correct temperature from the isopentane method was avoided.

Improving muscle biopsy protocols could lead to broader use of muscle samples to study human aging and other neuromuscular disease and increase use of muscle biopsy in clinical assessment of older adults with muscle weakness. The improvements in specimen quality and yield will limit patient burden and encourage multidimensional studies of muscle from histological assessments to genetic and biochemical analyses all done in the same sample to facilitate cross-correlation. Weaknesses to the VACORA System are primarily the cost, which is current ~$250 per needle. This cost is considerably more than that for the Bergstrom needle, but less than that for open biopsies despite being able to provide similar amounts of tissue.

## Conclusions

We find the VACORA Vacuum-Assisted Biopsy System to be very well suited for human muscle biopsies. Together with our simplified freezing procedure, we have seen improvements in our sample throughput and downstream analyses. The simplification and consistency of these methods allows for easier training of ancillary staff and will likely aid in dissemination of these protocols and the use of human muscle biopsies in basic science and translational research, and clinical trials.

## Abbreviations

UCH: University College Hospital
CDMD: Center for Duchenne Muscular Dystrophy
SEM: Standard Error of the Mean
